# A New Labeling Method of ^99m^ Tc-PSMA-HBED-CC

**DOI:** 10.1055/s-0045-1809921

**Published:** 2025-06-26

**Authors:** Benchamat Phromphao, Shuichi Shiratori

**Affiliations:** 1 Medical Physics Program, Department of Radiology, Faculty of Medicine, Chulalongkorn University, Bangkok, Thailand; 2 Chulalongkorn University Biomedical Imaging Group, Department of Radiology, Faculty of Medicine, Chulalongkorn University, Bangkok, Thailand; 3 Division of Nuclear Medicine, Department of Radiology, Faculty of Medicine Siriraj Hospital, Mahidol University, Bangkok, Thailand

**Keywords:** prostate cancer, PSMA-HBED-CC, PSMA-11, technetium-99m, ^99m^ Tc-PSMA-HBED-CC

## Abstract

**Objective:**

^68^ Ga-PSMA-HBED-CC ( ^68^ Ga-PSMA-11) was approved by the U.S. Food and Drug Administration as the first prostate-specific membrane antigen (PSMA)-targeted positron emission tomography (PET) imaging drug for patients with prostate cancer. However, the utility of ^68^ Ga-PSMA-HBED-CC may be limited due to PET/CT or PET/MR accessibility and ^68^ GaCl _3_ availability produced from ^68^ Ge/ ^68^ Ga generator or cyclotron. Thus, in-house preparation of ^99m^ Tc-PSMA-HBED-CC was developed as an alternative to ^68^ Ga-PSMA-HBED-CC to be ubiquitous and affordable in the worldwide population.

**Methods:**

A solution of ^99m^ Tc-pertechnetate was added to PSMA-HBED-CC and 4% SnCl _2_ ·2H _2_ O in a 10-mL sterile vial. The mixture was heated at 100°C for 15 minutes and then allowed to cool to room temperature. Labeling conditions were optimized to maximize the radiochemical yield of ^99m^ Tc-PSMA-HBED-CC. The chelation completeness was monitored using instant thin layer chromatography, and the stability of ^99m^ Tc-PSMA-HBED-CC was subsequently evaluated.

**Results:**

The radiolabeling of ^99m^ Tc-PSMA-HBED-CC was successful using the appropriate amount of 10 µg PSMA-HBED-CC 3 µg SnCl _2_ ·2H _2_ O and ^99m^ Tc-pertechnetate 370 MBq at 100°C for 15 minutes, yielded the best result in high radiochemical yield (71.49 ± 2.42%), radiochemical purity (98.29 ± 2.65%), and specific activity of 37.84 ± 1.47 GBq/µmol. ^99m^ Tc-PSMA-HBED-CC is stable with radiochemical purity of more than 95% within 4 hours at room temperature.

**Conclusion:**

A new labeling method of ^99m^ Tc-PSMA-HBED-CC was developed. Quality control parameters of ^99m^ Tc-PSMA-HBED-CC met the criteria in accordance with the European Pharmacopoeia.

## Introduction

Prostate cancer is the most common malignancy found in men and the second leading cause of cancer death worldwide. 1 Over the past two decades, the initial diagnosis and follow-up have been serum prostate-specific antigen levels, digital rectal examination, and some conventional imaging techniques including ultrasound, computed tomography, magnetic resonance imaging, and bone scintigraphy, but none provides highly specific and sensitive detection. Although transrectal ultrasound-guided prostate biopsy is currently accepted as the gold standard to provide the histopathological diagnosis of prostate cancer, 2 it is an invasive procedure that resulted in a risk of side effects and the accuracy of diagnosis. The accurate definition of tumor burden and its staging is particularly important for effective treatment selection. Therefore, molecular imaging, a noninvasive method, has been employed to visualize the tumor in both soft tissue and bone with higher specific and sensitive detection, monitored response to therapy to improve management of prostate cancer, clinical outcome, and patient's quality of life.

The Food and Drug Administration (FDA) approved ^68^ Ga-PSMA-HBED-CC ( ^68^ Ga-PSMA-11, previous name: ^68^ Ga-DKFZ-PSMA-11, generic name: ^68^ Ga-Gazetotide) as the first prostate-specific membrane antigen (PSMA)-targeted positron emission tomography (PET) imaging drug for men with prostate cancer. 3 The development of ^68^ Ga-PSMA-HBED-CC, which targets PSMA, has offered new perspectives for prostate cancer diagnosis and evaluation of therapeutic response. 4 PSMA is a cell surface transmembrane protein with 750 amino acids type II glycoprotein that primarily expresses in normal prostate epithelium and is overexpressed in prostate cancer cells including bone metastasis. 5 X-ray crystal structure analysis of PSMA, also known as *N* -acetylated *L* -aspartyl- *L* -glutamate peptidase (NAALADase I), has identified the critical interaction of potent inhibitors within the hydrophobic active site of the enzyme. 6 Consequently, several classes of NAALADase I inhibitors had been exploited for structure-based design platforms, leading to the novel synthesis of PSMA-HBED-CC. 7 8 9 To date, ^68^ Ga-PSMA-HBED-CC has explicitly demonstrated superior detection of PSMA-positive prostate cancer lesions in recurrent and metastatic sites over conventional imaging methods 10 11 and two other FDA-approved PET tracers, ^18^ F-fluciclovine and ^11^ C-choline that are used in patients with suspected cancer recurrence. 12 Recently, the FDA also approved ^18^ F-piflufolastat as the second PSMA-targeted PET imaging drug with prostate cancer.

Besides ^68^ Ga-PSMA-HBED-CC, the preferential use of ^99m^ Tc-labeled urea-based PSMA inhibitor has received interest as an alternative option to widespread the advantages of PSMA imaging due to a number of prostate cancer patients who are scheduled on PSMA imaging. The hybrid modality of single-photon emission CT (SPECT)/CT offers a wide range of workhorses in nuclear medicine with lower financial access, especially the remote medical center in which PET/CT facility is not available. Although the spatial resolution of ^99m^ Tc is not as good as that of ^68^ Ga, ^99m^ Tc provides a sufficiently long half-life of 6 hours in both preparation and accumulation in the target site. Moreover, the decay range of ^99m^ Tc is short enough to minimize radiation exposure to patients and medical staff.

**Fig. 1:**
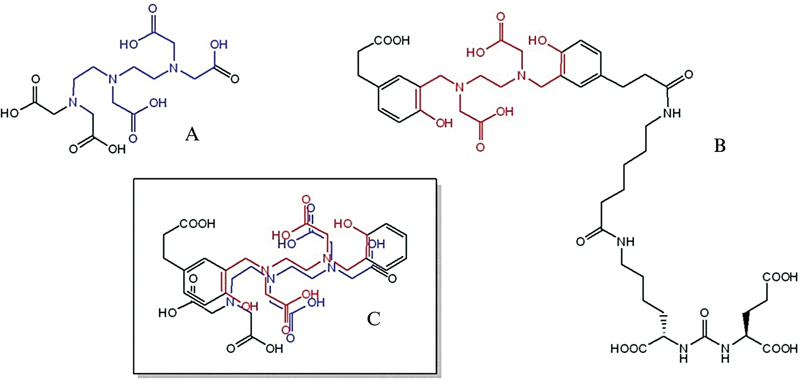
Chemical structures of ( **A** ) diethylenetriamine pentaacetic acid (DTPA), ( **B** ) PSMA-HBED-CC, and ( **C** ) imposed chelating motif.

While some ^99m^ Tc-labeled PSMA tracers have been previously reported using various PSMA ligands with several forms of complexation, for example, [ ^99m^ Tc(CO) _3_ (L) _3_ ] ^+^ , 13 14 15 MAG3-based ^99m^ Tc-PSMA-I&S, 16 ^99m^ Tc-MIP, 17 18 19 20 ^99m^ Tc-HYNIC-PSMA, 21 22 peptide-chelator-based ^99m^ Tc-DUPA, 23 ^99m^ Tc-PSMA-T4, 24 ^99m^ Tc-PSMA-tricarbonyl-HBED-CC, 25 and ^99m^ Tc-PSMA-HBED-CC, 26 it challenges to develop a convenient labeling method in a single step without coligand for ^99m^ Tc-complexation. According to our experience in theranostics, we adapted the routine standard labeling procedure of ^68^ Ga-PSMA-HBED-CC with the rationale that HBED-CC would serve as a suitable chelator in a mimic manner to diethylenetriamine pentaacetic acid (DTPA) as shown in Fig. 1 . Our attention focused on optimizing the labeling parameters to improve the radiosynthesis of ^99m^ Tc-PSMA-HBED-CC.

## Material and Methods

PSMA-HBED-CC was purchased from ABX advanced biochemical compounds (GmbH, Germany). Sodium ^99m^ Tc-pertechnetate was purchased from Global Medical Solution (Thailand). Stannous chloride dihydrate and hydrochloric acid were purchased from Sigma-Aldrich (Germany). The stock solution of 4% stannous chloride was freshly prepared and kept in a refrigerator. All chemicals and solvents were used without further purification unless otherwise noted. The C18 cartridge (Sep-Pak Light, lot no. 045732200A) was purchased from Waters (United States). The TLC scanner Raytest model MiniGita was used.

### Preparation of 4% Stannous Chloride Stock Solution

SnCl _2_ ·2H _2_ O 0.144 mg was added to 37% HCl 0.75 mL, followed by heating at 100°C for 5 minutes. After cooling down to room temperature, 6 N HCl 2.25 mL was added to 4% SnCl _2_ ·2H _2_ O, which should be freshly prepared before labeling.

### Labeling of PSMA-HBED-CC with Tc-99m

To a mixture of 4% SnCl _2_ ·2H _2_ O solution calculated as an amount of SnCl _2_ and an aliquot of PSMA-HBED-CC (10 µg in H _2_ O 100 µL) in 10 mL sterile vial, ^99m^ Tc-pertechnetate 370 MBq was added. The labeling was performed at 100°C for 15 minutes in a heating block, followed by a 10-minute cool down to reach room temperature. The crude product was passed through a C18 cartridge. ^99m^ Tc-PSMA-HBED-CC was slowly purged from a C18 cartridge using EtOH:H _2_ O (1:1) 2 mL to the final product vial.

### Quality Control

Radiochemical purity (RCP) analyses were performed using instant thin-layer chromatography (iTLC) on silica paper strips as a stationary phase with two different mobile phases. The free form of ^99m^ TcO _4_^−^ was determined using 0.9% normal saline, whereas ^99m^ Tc-colloid formation was determined using acetone as the mobile phase. The radioactivity distribution on iTLC strips was also determined on a TLC scanner. Subsequently, radiochemical yield (RCY) was calculated. The pH of the final product was determined using a pH indicator.

### Stability Test

The chemical stability of ^99m^ Tc-PSMA-HBED-CC was carried out by incubating the final product radioactivity in samples range 10.22 ± 0.40 mCi at room temperature for 6 hours and monitored by iTLC every hour. The radioconcentration used for the stability test was 2.04 mCi/mL. No stabilizer was added.

## Results

### Labeling of ^99m^ Tc-PSMA-HBED-CC

The labeling parameters were investigated to achieve the highest possible RCY. The quantity of PSMA-HBED-CC used in each experiment remained constant at 10 µg (0.011 µmol). In accordance with the DTPA cold kit formulation, the appropriate radioactivity of ^99m^ Tc-pertechnetate was determined in direct correlation with approximate 370 MBq (10 mCi), while maintaining the solution's pH at 5.0, as indicated in Table 1 .

**Table 1:** Labeling parameters in ^99m^ Tc-PSMA-HBED-CC

Iteration	SnCl _2_ ·2H _2_ O (µg)	^99m^ TcO _4_^−^ (mCi)	Labeling condition	Free form of ^99m^ TcO _4_^−^ (%)	^99m^ Tc-colloid (%)	RCY (%)
**1**	0.5	10.02	Room temperature 15 min	99.58	0.17	0.25
**2**	0.5	10.32	100°C, 15 min	88.89	0.00	11.11
**3**	1.0	9.97	100°C 15 min	81.82	0.00	18.18
**4**	2.0	10.16	100°C, 15 min	64.29	10.00	25.71
**5**	2.5	10.61	100°C, 15 min	20.00	20.00	60.00
**6**	3.0	11.02	100°C, 15 min	9.09	25.00	65.91
**7**	3.5	11.64	100°C, 15 min	0.00	41.67	58.33
**8**	4.0	11.15	100°C, 15 min	0.00	50.00	50.00

Abbreviation: RCY, radiochemical yield.

In the preliminary experiment, 10.02 mCi of ^99m^ TcO _4_^−^ was combined with 0.5 µg of SnCl _2_ at room temperature for 15 minutes that resulted in a RCY of 0.25%, with 99.58% of ^99m^ TcO _4_^−^ remaining unbound. Subsequently, the reaction temperature was increased to 100°C for 15 minutes, in alignment with the standard procedure for labeling ^68^ Ga-PSMA-HBED-CC. This adjustment resulted in a RCY of 11.11%, affirming the choice to set the reaction conditions at 100°C for 15 minutes. In iterations 3 to 8, the amount of SnCl _2_ was progressively adjusted to enhance the RCY. By increasing the SnCl _2_ quantity by 0.5 µg in each iteration, the highest RCY of 65.91% was achieved in the sixth iteration. However, when the amount of SnCl _2_ exceeded 3.5 µg, complete chelation occurred between PSMA-HBED-CC and ^99m^ TcO _4_^−^ , which also led to a significant rise in the formation of hydrolyzed species, ultimately diminishing the RCY. Total radioactivities of the final product recovered after labeling are 10.12 ± 0.58 mCi.

### Chemical Stability of ^99m^ Tc-PSMA-HBED-CC

**Fig. 2:**
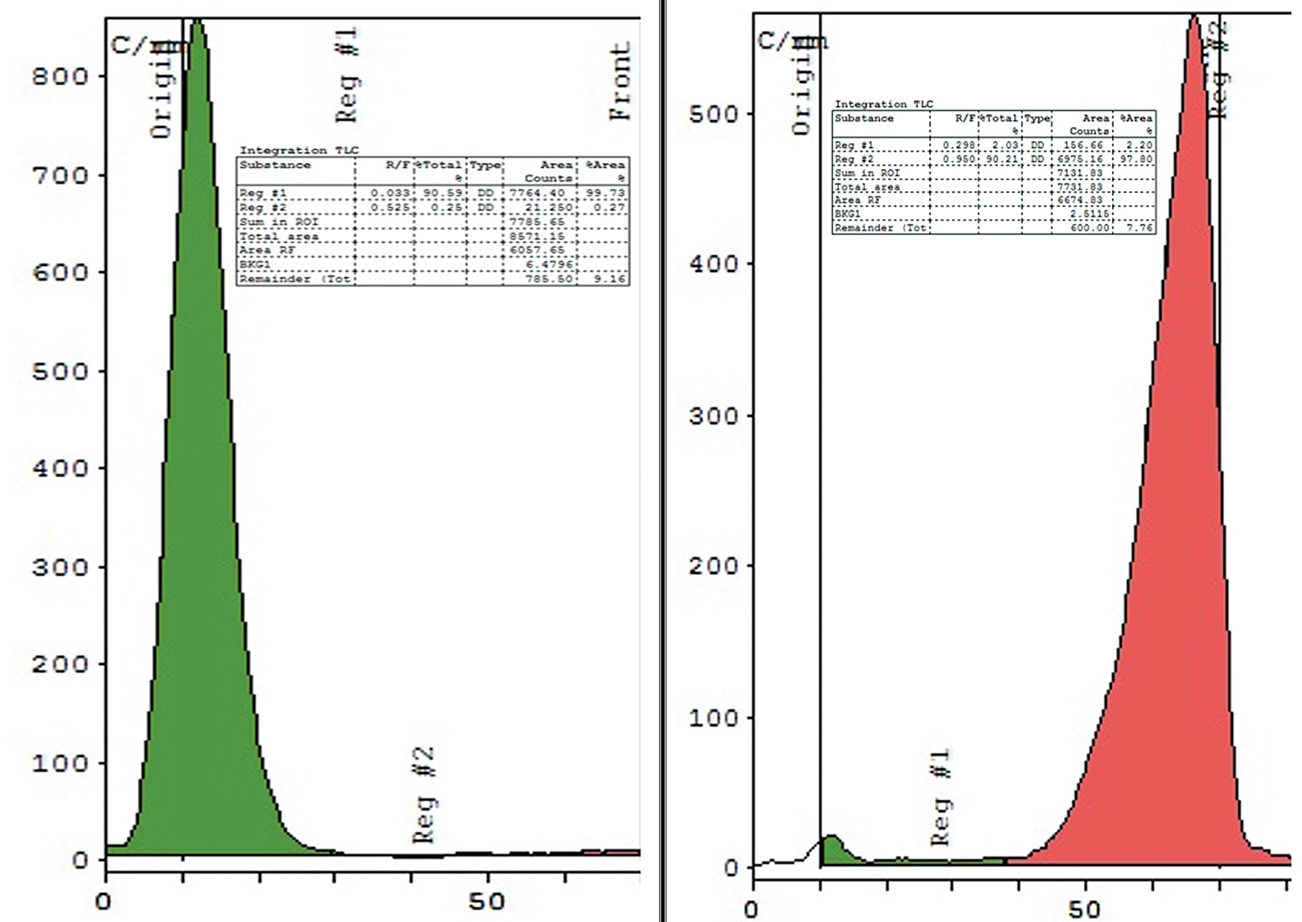
Instant thin-layer chromatography (iTLC) chromatogram of ^99m^ Tc-PSMA-HBED-CC; (left) in acetone as mobile phase, (right) in 0.9% normal saline as mobile phase.

**Fig. 3:**
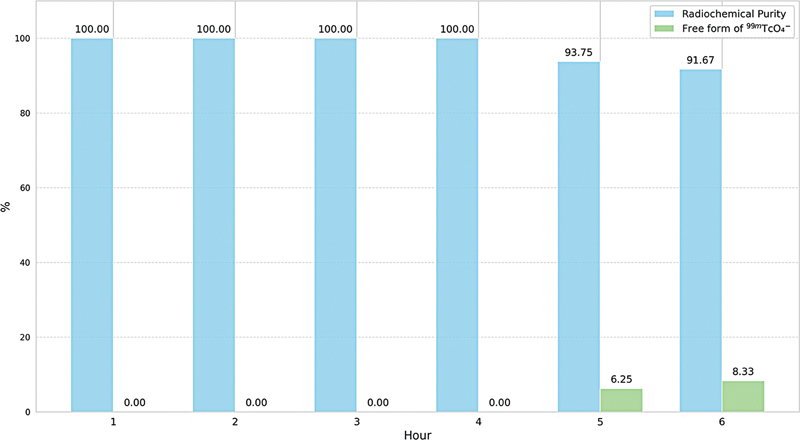
Chemical stability of ^99m^ Tc-PSMA-HBED-CC at room temperature.

The chemical stability of ^99m^ Tc-PSMA-HBED-CC was evaluated using the optimized labeling method in the 6th iteration that produced the highest RCY. The compound was incubated at room temperature for 6 hours, and RCP was checked hourly through iTLC ( Fig. 2 ). The findings are shown in Fig. 3 . In general, the RCP of Tc-99m radiopharmaceuticals should meet or exceed 95%. This study demonstrated that the RCP of ^99m^ Tc-PSMA-HBED-CC remained above 95% for up to 4 hours after labeling, and stayed above 90% at the 5th and 6th hours.

## Discussion

Since the identification of PSMA as an antigen and the discovery of the specific antibody 7E11-C5 (capromab) for both normal and malignant prostate epithelium, as reported by Horoszewicz et al, 27 PSMA has emerged as a crucial target for prostate cancer cells. 28 29 30 In 2012, Eder et al developed the urea-based PET tracer ^68^ Ga-PSMA-HBED-CC (formerly ^68^ Ga-DKFZ-PSMA-11) to address the limitations of the lead antibody J591. 31 The U.S. FDA approved ^68^ Ga-PSMA-HBED-CC as the first PET imaging agent for PSMA-positive lesions in men with prostate cancer in December 2020 3 and ^18^ F-piflufolastat as the second PET imaging agent for PSMA-positive lesions in men with prostate cancer in May 2021. 3 Inspired by this breakthrough, our study aimed to develop ^99m^ Tc-PSMA-HBED-CC as a SPECT imaging analog to increase accessibility for prostate cancer diagnosis.

PSMA-HBED-CC was chosen for this study due to its widespread clinical use and commercial availability. The dose of PSMA-HBED-CC was standardized at 10 μg. To optimize the manual labeling of ^99m^ Tc-PSMA-HBED-CC, various labeling conditions and amounts of calculated SnCl _2_ were assessed. The results, presented in Table 1 , indicate that ^99m^ Tc-PSMA-HBED-CC is a thermodynamically favorable product. Using 2.5 to 4.0 μg of SnCl _2_ resulted in a RCY exceeding 50%. When the SnCl _2_ amount exceeded 3.5 μg, no uncomplexed Tc-99m was detected, indicating effective reduction of TcO _4_^−^ . However, higher amounts of SnCl _2_ also increased colloid formation. Optimal quantitative radiolabelling of 10 μg of PSMA-HBED-CC was achieved with 3.0 μg of SnCl _2_ . To prevent colloid formation, both SnCl _2_ and PSMA-HBED-CC must be present in the reaction mixture before adding TcO _4_^−^ to form the desired complex.

The chemical stability of ^99m^ Tc-PSMA-HBED-CC was evaluated by incubating it at room temperature. As shown in Fig. 2 , it retained RCP above 95% for up to 4 hours. After this period, free Tc-99m increased. Therefore, it is recommended to use ^99m^ Tc-PSMA-HBED-CC within 4 hours of preparation or store it in a refrigerator to maintain stability.

Vats et al 26 previously reported the preparation of ^99m^ Tc-PSMA-HBED-CC using 50 mg of PSMA-HBED-CC, 40 mg of SnCl _2_ , and TcO _4_^−^ 740 MBq at pH 5, yielding a RCY of 60 ± 5%, a RCP greater than 98%, and specific activity of 15 ± 5 GBq/µmol. However, they did not conduct stability tests. Economically, our study used 10 µg of PSMA-HBED-CC, 3 µg of SnCl _2_ , and 370 MBq of ^99m^ Tc-pertechnetate at 100°C for 15 minutes, achieving a higher RCY (71.49 ± 2.42%), RCP (98.29 ± 2.65%), and specific activity (37.84 ± 1.47 GBq/µmol). This method is more cost-effective and easier to manipulate.

## Conclusion

To optimize the labeling of PSMA-HBED-CC with ^99m^ Tc-pertechnetate for prostate cancer imaging, the labeling procedure should be carried out at 100°C for 15 minutes using 3 µg of SnCl _2_ , minimizing the presence of free ^99m^ Tc-pertechnetate and colloid formation. Purification with a C18 cartridge is required to achieve RCP that complies with the European Pharmacopoeia standards. The stability of ^99m^ Tc-PSMA-HBED-CC remains robust for up to 4 hours at room temperature, with RCP exceeding 95%. It is recommended to use the labeled product within 4 hours of preparation.

## References

[1] Bray F Ferlay J Soerjomataram I Siegel R L Torre L A Jemal A Global cancer statistics 2018: GLOBOCAN estimates of incidence and mortality worldwide for 36 cancers in 185 countries CA Cancer J Clin 2018 68 06 394 424 30207593 10.3322/caac.21492

[2] Moe A Hayne D Transrectal ultrasound biopsy of the prostate: does it still have a role in prostate cancer diagnosis? Transl Androl Urol 2020 9 06 3018 3024 33457275 10.21037/tau.2019.09.37 PMC7807378

[3] Hennrich U Eder M [ ^68^ Ga] Ga-PSMA-11: the first FDA-approved ^68^ Ga-radiopharmaceutical for PET imaging of prostate cancer Pharmaceuticals 2021 14 08 713 725 34451810 10.3390/ph14080713 PMC8401928

[4] Afshar-Oromieh A Haberkorn U Eder M Eisenhut M Zechmann C M [ ^68^ Ga]Gallium-labelled PSMA ligand as superior PET tracer for the diagnosis of prostate cancer: comparison with _18_ F-FECH Eur J Nucl Med Mol Imaging 2012 39 06 1085 1086 22310854 10.1007/s00259-012-2069-0

[5] Silver D A Pellicer I Fair W R Heston W D Cordon-Cardo C Prostate-specific membrane antigen expression in normal and malignant human tissues Clin Cancer Res 1997 3 01 81 85 9815541

[6] Davis M I Bennett M J Thomas L M Bjorkman P J Crystal structure of prostate-specific membrane antigen, a tumor marker and peptidase Proc Natl Acad Sci U S A 2005 102 17 5981 5986 15837926 10.1073/pnas.0502101102 PMC556220

[7] Mesters J R Henning K Hilgenfeld R Human glutamate carboxypeptidase II inhibition: structures of GCPII in complex with two potent inhibitors, quisqualate and 2-PMPA Acta Crystallogr D Biol Crystallogr 2007 63(Pt 4):508 513 17372356 10.1107/S090744490700902X

[8] Lundmark F Olanders G Rinne S S Abouzayed A Orlova A Rosenström U Design, synthesis, and evaluation of linker-optimised PSMA-targeting radioligands Pharmaceutics 2022 14 05 1098 1117 35631684 10.3390/pharmaceutics14051098 PMC9147442

[9] Barinka C Byun Y Dusich C L Interactions between human glutamate carboxypeptidase II and urea-based inhibitors: structural characterization J Med Chem 2008 51 24 7737 7743 19053759 10.1021/jm800765e PMC5516903

[10] Maurer T Eiber M Schwaiger M Gschwend J E Current use of PSMA-PET in prostate cancer management Nat Rev Urol 2016 13 04 226 235 26902337 10.1038/nrurol.2016.26

[11] Virgolini I Decristoforo C Haug A Fanti S Uprimny C Current status of theranostics in prostate cancer Eur J Nucl Med Mol Imaging 2018 45 03 471 495 29282518 10.1007/s00259-017-3882-2 PMC5787224

[12] Li M Zelchan R Orlova A The performance of FDA-approved PET imaging agents in the detection of prostate cancer Biomedicines 2022 10 10 2533 2556 36289795 10.3390/biomedicines10102533 PMC9599369

[13] Banerjee S R Foss C A Castanares M Synthesis and evaluation of technetium-99m- and rhenium-labeled inhibitors of the prostate-specific membrane antigen (PSMA) J Med Chem 2008 51 15 4504 4517 18637669 10.1021/jm800111u PMC3336105

[14] Maresca K P Hillier S M Lu G Small molecule inhibitors of PSMA incorporating technetium-99m for imaging prostate cancer: effects of chelate design on pharmacokinetics Inorg Chim Acta 2012 389 168 175

[15] Lu G Maresca K P Hillier S M Synthesis and SAR of ^99m^ Tc/Re-labeled small molecule prostate specific membrane antigen inhibitors with novel polar chelates Bioorg Med Chem Lett 2013 23 05 1557 1563 23333070 10.1016/j.bmcl.2012.09.014

[16] Robu S Schottelius M Eiber M Preclinical evaluation and first patient application of ^99m^ Tc-PSMA-I&S for SPECT imaging and radioguided surgery in prostate cancer J Nucl Med 2017 58 02 235 242 27635024 10.2967/jnumed.116.178939

[17] Hillier S M Maresca K P Lu G ^99m^ Tc-labeled small-molecule inhibitors of prostate-specific membrane antigen for molecular imaging of prostate cancer J Nucl Med 2013 54 08 1369 1376 23733925 10.2967/jnumed.112.116624

[18] Vallabhajosula S Nikolopoulou A Babich J W ^99m^ Tc-labeled small-molecule inhibitors of prostate-specific membrane antigen: pharmacokinetics and biodistribution studies in healthy subjects and patients with metastatic prostate cancer J Nucl Med 2014 55 11 1791 1798 25342385 10.2967/jnumed.114.140426

[19] Reinfelder J Kuwert T Beck M First experience with SPECT/CT using a ^99m^ Tc-labeled inhibitor for prostate-specific membrane antigen in patients with biochemical recurrence of prostate cancer Clin Nucl Med 2017 42 01 26 33 27775936 10.1097/RLU.0000000000001433

[20] Schmidkonz C Goetz T I Kuwert T PSMA SPECT/CT with ^99m^ Tc-MIP-1404 in biochemical recurrence of prostate cancer: predictive factors and efficacy for the detection of PSMA-positive lesions at low and very-low PSA levels Ann Nucl Med 2019 33 12 891 898 31502084 10.1007/s12149-019-01400-6

[21] Zhang J Zhang J Xu X Evaluation of radiation dosimetry of ^99m^ Tc-HYNIC-PSMA and imaging in prostate cancer Sci Rep 2020 10 01 4179 4187 32144340 10.1038/s41598-020-61129-5 PMC7060171

[22] Xu X Zhang J Hu S ^99m^ Tc-labeling and evaluation of a HYNIC modified small-molecular inhibitor of prostate-specific membrane antigen Nucl Med Biol 2017 48 69 75 28273495 10.1016/j.nucmedbio.2017.01.010

[23] Kularatne S A Zhou Z Yang J Post C B Low P S Design, synthesis, and preclinical evaluation of prostate-specific membrane antigen targeted ^(99m)^ Tc-radioimaging agents Mol Pharm 2009 6 03 790 800 19361232 10.1021/mp9000712 PMC9190123

[24] Sergieva S Mangaldgiev R Dimcheva M Nedev K Zahariev Z Robev B SPECT-CT imaging with [ ^99m^ Tc] PSMA-T4 in patients with recurrent prostate cancer Nucl Med Rev 2021 24 02 70 81 10.5603/NMR.2021.0018 34382671

[25] Shi S Yao L Li L Synthesis of novel technetium-99m tricarbonyl-HBED-CC complexes and structural prediction in solution by density functional theory calculation R Soc Open Sci 2019 6 11 191247 191260 31827858 10.1098/rsos.191247 PMC6894603

[26] Vats K Agrawal K Sharma R Sarma H D Satpati D Dash A Preparation and clinical translation of ^99m^ Tc-PSMA-11 for SPECT imaging of prostate cancer Med Chem Commun 2019 10 12 2111 2117 10.1039/c9md00401g PMC7067243 32190233

[27] Horoszewicz J S Kawinski E Murphy G P Monoclonal antibodies to a new antigenic marker in epithelial prostatic cells and serum of prostatic cancer patients Anticancer Res 1987 7(5B):927 935 2449118

[28] Lopes A D Davis W L Rosenstraus M J Uveges A J Gilman S C Immunohistochemical and pharmacokinetic characterization of the site-specific immunoconjugate CYT-356 derived from antiprostate monoclonal antibody 7E11-C5 Cancer Res 1990 50 19 6423 6429 1698122

[29] Kahn D Williams R D Seldin D W Radioimmunoscintigraphy with 111indium labeled CYT-356 for the detection of occult prostate cancer recurrence J Urol 1994 152(5 Pt 1):1490 1495 7523704 10.1016/s0022-5347(17)32453-9

[30] Wynant G E Murphy G P Horoszewicz J S Immunoscintigraphy of prostatic cancer: preliminary results with ^111^ In-labeled monoclonal antibody 7E11-C5.3 (CYT-356) Prostate 1991 18 03 229 241 2020619 10.1002/pros.2990180305

[31] Eder M Schäfer M Bauder-Wüst U ^68^ Ga-complex lipophilicity and the targeting property of a urea-based PSMA inhibitor for PET imaging Bioconjug Chem 2012 23 04 688 697 22369515 10.1021/bc200279b

